# Development and characterisation of a 3D multi-cellular *in vitro* model of normal human breast: a tool for cancer initiation studies

**DOI:** 10.18632/oncotarget.3803

**Published:** 2015-04-12

**Authors:** Claire E. Nash, Georgia Mavria, Euan W. Baxter, Deborah L. Holliday, Darren C. Tomlinson, Darren Treanor, Vera Novitskaya, Fedor Berditchevski, Andrew M. Hanby, Valerie Speirs

**Affiliations:** ^1^ Leeds Institute of Cancer and Pathology, University of Leeds, Leeds, UK; ^2^ Leeds Institute of Biomedical and Clinical Sciences, University of Leeds, Leeds, UK; ^3^ Leeds Teaching Hospitals NHS Trust, Leeds, UK; ^4^ School of Cancer Sciences, University of Birmingham, Birmingham, UK; ^5^ Current address: The Research Institute of the McGill University Health Centre, Montreal, Quebec, Canada

**Keywords:** 3D cell culture, breast, HER2

## Abstract

Multicellular 3-dimensional (3D) *in vitro* models of normal human breast tissue to study cancer initiation are required. We present a model incorporating three of the major functional cell types of breast, detail the phenotype and document our breast cancer initiation studies. Myoepithelial cells and fibroblasts were isolated and immortalised from breast reduction mammoplasty samples. Tri-cultures containing non-tumorigenic luminal epithelial cells HB2, or HB2 overexpressing different HER proteins, together with myoepithelial cells and fibroblasts were established in collagen I. Phenotype was assessed morphologically and immunohistochemically and compared to normal breast tissue. When all three cell types were present, polarised epithelial structures with lumens and basement membrane production were observed, akin to normal human breast tissue. Overexpression of HER2 or HER2/3 caused a significant increase in size, while HER2 overexpression resulted in development of a DCIS-like phenotype. In summary, we have developed a 3D tri-cellular model of normal human breast, amenable to comparative analysis after genetic manipulation and with potential to dissect the mechanisms behind the early stages of breast cancer initiation.

## INTRODUCTION

Researchers are beginning to appreciate the need for more complex multicellular three-dimensional (3D) laboratory models in order to study the processes associated with disease progression. In recent years there has been a shift towards developing 3D *in vitro* models that better represent breast tissues. These typically involve culturing breast epithelial cells alone or with fibroblasts [1-3] in 3D in the presence of extracellular matrix (ECM) such as Matrigel^TM^ or collagen I. Mammary luminal epithelial cells respond to ECM by forming acini-like structures [4]. By culturing cells with specific ECM constituents, cell-cell and cell-ECM interactions can be represented in the laboratory [5]. This has led to a better understanding of how different breast cell types interact with each other and the surrounding ECM as well as identification of key intercellular signalling proteins involved in maintaining luminal cell polarity such as integrins [6] and cadherins [7].

Current 3D *in vitro* models of normal human breast are limited. Compared to the wealth of breast cancer cell lines available to represent the molecular subtypes of breast cancer [8], normal breast epithelial cell lines are scarce. MCF10A is the most commonly used cell line to represent normal breast. When cultured in Matrigel^TM^, polarised acini-like structures with hollow lumens develop which produce basement membrane proteins [9, 10]. These models often incorporate fibroblasts [1-3]. However, MCF10A cells have a propensity to adopt a basal phenotype [11, 12] and are sensitive to changes in culture conditions [13] making them challenging to work with. Furthermore there are limitations to using Matrigel^TM^; although used widely, the levels of growth factors and basement membrane components are heterogeneous [14] and the substance itself is prepared from Engelbreth-Holm-Swarm mouse sarcoma cells. The main constituent of breast stroma is collagen I [15], a regulator of morphology and phenotype of breast epithelial cells [1, 16, 17]. Moreover, variation in the amount of collagen and subsequent mammographic density may be linked to the onset of breast cancer [18, 19]. Thus the case for incorporating collagen I in the development of 3D *in vitro* models of breast tissue is strong.

In addition to the role fibroblasts play on regulating luminal epithelial behaviour [20-22], breast myoepithelial cells are important in maintaining luminal epithelial polarisation and architecture [23, 24]. These are considered tumour suppressive [25, 26], expressing a number of tumour suppressor proteins e.g. maspin and ERβ, structural ECM proteins e.g. fibronectin, proteinase inhibitors e.g. tissue inhibitor of metalloproteinase-1 (TIMP1) and angiogenic inhibitors e.g. thrombospondin-1 [25, 27, 28]. However, studies incorporating these cells into 3D models are limited.

This study aimed to create *in vitro* a 3D tri-culture system of normal human breast that incorporates an alternative normal luminal epithelial cell line HB2 [29, 30], together with myoepithelial and fibroblast cells in a physiologically relevant matrix, collagen I. We demonstrated that the model is representative of normal human breast by comparing the morphology and immunoprofile to ‘gold standard’ normal human breast tissue sections, and determined that the model was amenable to cancer initiation studies through HER overexpression in luminal epithelial cells.

## RESULTS

### 3D *in vitro* tri-culture model recapitulates the morphology and phenotype of normal human breast tissue

**Figure 1:**
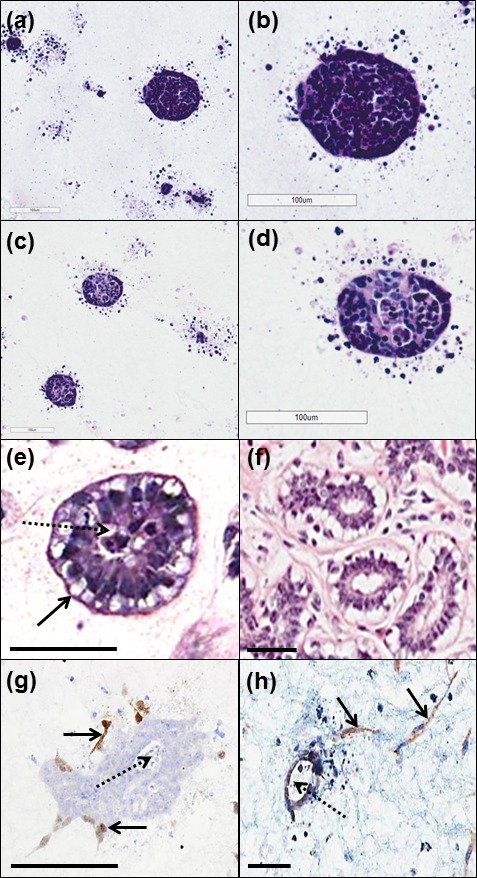
Comparison of morphology of 3D tri-culture model with normal human breast tissue When cultured alone (**a**, **b**), HB2 cells formed tight spherical units of varying size with occasional scattered cells. A similar phenotype was observed when HB2 were co-cultured with Myo1089 cells (**c**, **d**). In tri-cultures containing HB2, Myo1089 and fibroblasts, ductal structures with hollow lumens (dotted arrow) were observed surrounded by a border of clear myoepithelial cells (arrow; **e**) akin to normal human breast acini (**f**). Immunohistochemical characterisation using tGFP and dsRed antibodies confirmed the distribution of myoepithelial cells at the outer edges of HB2 structures with hollow lumens (dotted arrow) (arrows; **g**) and fibroblasts loosely distributed throughout collagen gels (arrows; **h**), respectively. Scale bars = 100 μm.

HB2 cells were cultured alone, with Myo1089 cells or with Myo1089 and fibroblasts in collagen I gels and phenotype assessed by H&E after 21 days. When cultured alone, HB2 cells formed tight spherical units of varying size with occasional scattered cells (Figure 1a, 1b). A similar phenotype was observed when HB2 were co-cultured with Myo1089 cells (Figure 1c, 1d). Tri-culture of HB2, Myo1089 and fibroblasts, resulted in the arrangement of Myo1089 myoepithelial cells around the outer edges of spherical lumen containing HB2 epithelial cells with fibroblasts loosely distributed throughout the collagen gel (Figure 1e) akin to normal breast acini (Figure 1f). This phenotype was only observed when all 3 cell types were present. Spatial localisation of myoepithelial cells and fibroblasts were confirmed by immunohistochemistry (Figure 1g, 1h), respectively.

**Figure 2:**
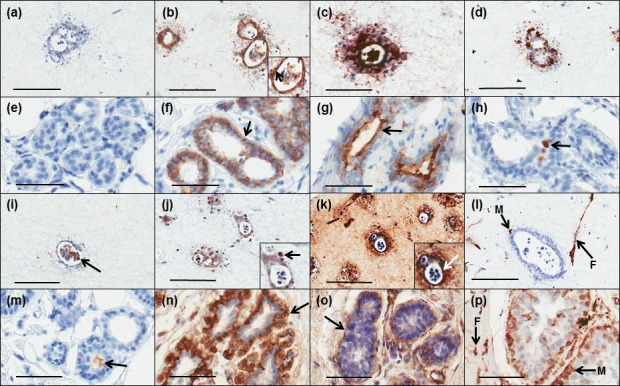
Comparison of immunohistochemical profiles of 3D tri-cultures with those of normal breast tissue The phenotype of 3D *in vitro* tri-cultures (**a**-d; i-l) was assessed by immunohistochemistry and compared to cross sections of breast reduction mammoplasty tissue (e-h; m-p). Negative controls (**a**, **e**). E-cadherin was expressed by HB2 epithelial cells at cell-cell junctions (b, arrow) similar to luminal epithelium of breast acini (f, arrow). EMA expression was concentrated at the HB2 cell-lumen interface in co-units (**c**) and was specific to apical membranes of luminal epithelium of breast acini (g, arrow). The majority of cells within HB2 co-units expressed Ki67 (**d**) with scattered positivity within breast acini (**h**). M30 staining demonstrated apoptosis within lumens of HB2 co-units (i, arrow) and also in breast acini (m, arrow). α-SMA was restricted to myoepithelial cells on the periphery of HB2 co-units (j, arrow) while a continuous layer of α-SMA positive myoepithelial cells surround luminal epithelium of breast acini (n, arrow). Collagen IV was concentrated around outside edges of HB2 co-units (k, arrow) and similarly is demonstrated encapsulating breast acini (o, arrow). Vimentin was expressed in both Myo1089 cells (M) and fibroblasts (F) in the tri-culture model (**l**) analogous to Vimentin positivity in myoepithelial cells (M) and fibroblasts (F) in normal breast tissue (**p**). Original magnification for tri-culture model = 20x; for normal tissue = 40x.

We then conducted immunohistochemical characterisation of 3D *in vitro* tri-cultures and compared this to normal human breast tissue. As shown in Figure 2, tri-culture phenotype (a-d, i-l) was strikingly similar to that of normal breast tissue (e-h, m-p). Controls (no primary antibody) are shown in Figure 2a and 2i. E-cadherin (E-cad) was expressed at cell-cell junctions (Figure 2b, 2f) with EMA at apical membranes (Figure 2c, 2g). Some cell proliferation was identified by Ki67 staining (Figure 2d, 2h), with apoptosis detected within central lumens by M30 staining (Figure 2i, 2m). α-SMA was specific to myoepithelial cells (Figure 2j, 2n) while basement membrane production was detected around outer edges of breast ducts by collagen IV expression (Figure 2k, 2o). Vimentin expression was detected in both myoepithelial cells and fibroblasts (Figure 2l, 2p). Comparison of these immunohistochemical features to normal breast tissue sections demonstrated strikingly strong similarities.

### Overexpression of HER proteins affect the morphology and phenotype of the tri-culture model

**Figure 3:**
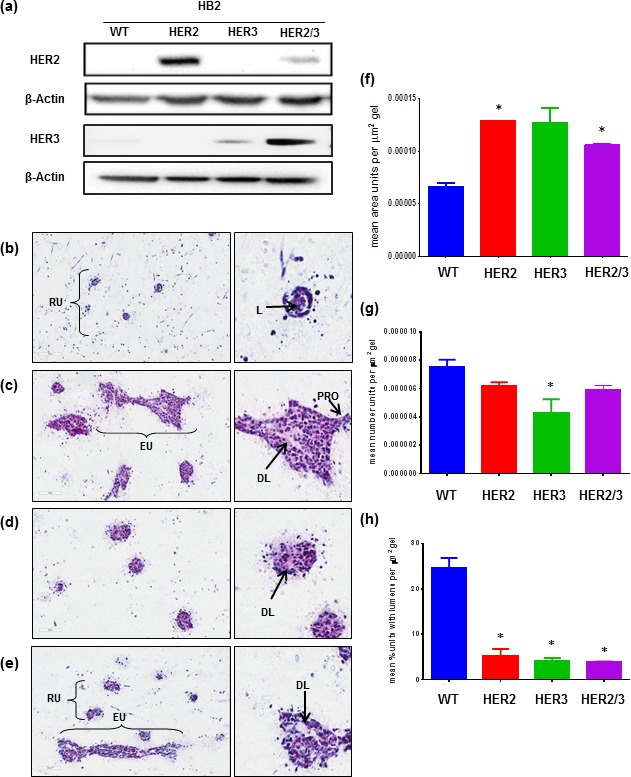
Overexpression of HER proteins in HB2 cells affects 3D architecture Stable overexpression of HER2 and HER3 in HB2 cells was shown by western blot (**a**). WT cells in 3D tri-culture (**b**) formed rounded units (RU) with lumens (L). When HER2 was overexpressed (**c**), formation of much larger elongated units (EU) with distorted lumens (DL) and protrusions (PRO) from edges was observed. HER3 overexpression (**d**) resulted in rounded units with distorted lumens (DL) while overexpression of HER2 and HER3 cells (**e**) resulted in a combination of rounded units (RU) and elongated units (EU) with distorted lumens (DL). Quantitative analysis confirmed HER2 and HER2/3 overexpression induced significantly larger HB2 unit formation (**f**). Only HER3 overexpression alone significantly altered the number of HB2 units formed (**g**) while HER2, HER3 and HER2/3 overexpression significantly reduced lumen formation (**h**). Bars denote mean from three gel replicates and error bars standard error of the mean (SEM). * = *p* < 0.05.

To test whether the tri-culture model was suitable for cancer initiation studies, HER2 and HER3 proteins were overexpressed in HB2 cells. Protein presence was confirmed by western blot analysis (Figure 3a). Compared to controls (Figure 3b) upon HER2 overexpression, HB2 cells formed larger, elongated units with distorted lumens and the appearance of protrusions from outer edges (Figure 3c). HER3 overexpressing cells also formed larger units containing distorted lumens but retained a spherical shape (Figure 3d). HB2 cells overexpressing both HER2 and HER3 formed a combination of smaller rounded units and larger elongated units that were larger than HB2 controls and also featured distorted lumens (Figure 3e). Quantitative analysis supported these observations. HB2 co-units significantly increased in size following HER2, or HER2/3 overexpression (Figure 3f, *p* = <0.05). Overexpression of HER3 induced a significant decrease (Figure 3g, *p* = 0.0442) in co-unit formation, while overexpression of HER2, HER3 and HER2/3 caused a decrease in lumen formation compared to HB2 controls (Figure 3h, *p* = <0.05). To examine if all 3 cell types were necessary to mediate these phenotypic changes, HER overexpressing HB2 cells were co-cultured with either myoepithelial cells or fibroblasts. Co-culture with myoepithelial cells had an organisational effect inducing the formation of rounded cohesive units while co-culture with fibroblasts had the opposite effects, resulting in larger co-units with a discohesive and disorganised appearance (Suppl. Figure 3).

### HER2 overexpressing HB2 3D tri-cultures resemble human DCIS phenotype

**Figure 4:**
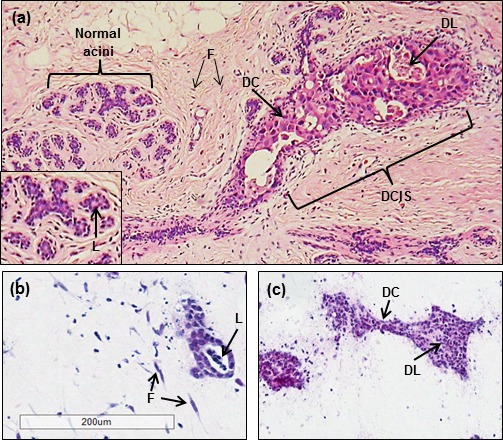
Comparison of HER2 overexpressing tri-cultures to human breast tissue containing DCIS A cross section of human breast tissue (**a**) shows normal acini which are small and rounded with cleared central lumens (L, inset). Areas of DCIS consist of elongated discohesive cells (DC) with distorted lumens containing necrotic cells (DL). Co-units of the tri-culture model are also small and rounded with central lumens (L), strongly resembling normal breast acini. Overexpression of HER2 in luminal epithelium (**c**) resulted in an elongated, branched shape, the appearance of distorted lumen with possible necrosis (DL) and also discohesive cells (DC), reminiscent of a DCIS-like phenotype. Original magnification x20 (tri-culture model); 40x (breast tissue).

H&E stained sections of HER2 overexpressing and HB2 control 3D tri-cultures were compared with a cross section of a human breast tissue that contained areas of both DCIS and normal adjacent acini (Figure 4a). As previously, HB2 control units were small, rounded, contained lumens (Figure 4b) and were similar phenotypically to normal breast acini (Figure 4a). In the breast tissue section, areas of DCIS featured large elongated ducts and contained distorted lumens with necrotic tissue (Figure 4a). These also contained enlarged and discohesive cells. HER2 overexpressing HB2 units were similarly elongated in shape, containing discohesive cells with distorted luminal spaces thought to be a product of necrosis (Figure 4c). Immunohistochemical characterisation showed no change in Ki67 expression between HB2 control units or those containing HER2-overexpressing cells, however a reduction in M30 and E-cadherin was observed (Suppl. Figure 4), suggesting these morphological changes are a result of apoptosis and loss of cell adhesion. This not only confirmed that the normal 3D tri-culture model was robust and retained features of normal breast acini, but also that morphological and phenotypical features of DCIS could be recapitulated through overexpression of HER2 in the 3D tri-culture model.

## DISCUSSION

With scientists now recognising the importance of the microenvironment in cancer initiation and progression, there is an increased need for robust 3D *in vitro* models which incorporate components of the microenvironment. Here, we present, a 3D *in vitro* model of normal human breast that incorporates not only breast luminal epithelium with fibroblasts, but also myoepithelial cells all embedded in the physiologically relevant matrix collagen I. This model reflects the morphology and phenotype of normal breast providing a novel tool to investigate the mechanisms behind the early events of cancer initiation and the formation of pre-malignant lesions.

Following optimisation of culture conditions including culture medium, cell numbers and duration of culture (data not shown), we demonstrated that co-culturing the three major cell types comprising the mammary duct in collagen I resulted in the arrangement of a border of myoepithelial cells surrounding HB2 epithelial cells which formed ductal structures with hollow lumens, with fibroblasts loosely distributed throughout the collagen gel. This morphology was remarkably similar to ‘gold standard’ normal human breast acini. It was necessary to use HB2 cells instead of the more commonly used MCF10A cells as we found that, as others have reported [11, 12], the latter exhibited plasticity, with evidence of at least 2 different sub-populations particularly when these were incorporated into more complex multicellular 3D collagen I models (Nash et al., manuscript in preparation).

This was confirmed further by immunohistochemical analysis of a panel of breast epithelial and mesenchymal markers and expression profiles compared to normal breast tissue. Near-identical expression patterns provided confidence that our 3D *in vitro* tri-culture model was a robust model of normal human breast tissue. Deposition of basement membrane protein collagen IV around the outer edges of co-units was observed, supporting previous reports that inclusion of myoepithelial cells with luminal epithelial cells in 3D collagen I matrix can abrogate the need for basement membrane preparations such as Matrigel^TM^ [24] due to their ability to produce proteins required for basement membrane formation [25]. All co-units displayed E-cadherin expression between HB2 cell junctions with epithelial membrane antigen situated at the apical-luminal interface of HB2 cells indicating the polarisation of HB2 cells. The presence of lumens cleared via apoptosis as detected by M30 expression. In addition, we proved that each cell type retained a phenotype representative of their corresponding cell type in normal breast tissue with vimentin expression detected in Myo1089 and fibroblasts and α-smooth muscle actin found in Myo1089 cells only. Continued correlation to normal human breast tissue supports the validity of the tri-cultures as models of normal breast acini.

We then sought to determine if the model could be used as a tool to study cancer initiation processes. Focussing on the luminal epithelium, we aimed to disrupt the normal architecture to produce a morphology and phenotype akin to early pre-malignant breast lesions, through overexpression of HER proteins. HER2 gene amplification and protein overexpression is observed in around 16% of breast cancers [31] and is associated with a poor prognosis and survival in breast cancer patients due to its relationship with more aggressive tumours [32]. HER2 overexpression also occurs in up to 50% of DCIS [33] suggesting a role in cancer initiation with murine studies demonstrating HER2 overexpression alone is sufficient to induce transformation of mammary epithelium in a single step [34, 35]. This demonstrates the potency of HER2 as an oncogene. The HER2-HER3 heterodimer is the most potent and mitogenic heterodimer of the HER family. The potential of the HER2-HER3 heterodimer to activate PI3K [36] and the MAPK pathway [37, 38] makes HER2 a key regulator of a variety of cell responses such as proliferation, apoptosis and differentiation. Our study demonstrated that overexpression of HER2 alone had the most dramatic effect on the architecture of co-units. These were significantly larger, more elongated and less cohesive, and featured protrusions similar to DCIS. Furthermore, lumen formation was significantly reduced within these co-units. Immunohistochemical characterisation suggested that these features may be attributed to apoptosis and a reduction in cell adhesion. Previous reports have proposed that HER2 overexpression in MCF10A cells in 3D Matrigel™ can result in evasion of apoptosis, lumen filling, disengagement of inner cell layers from the basement membrane, an increase in epithelial-mesenchymal transition and cell migration, and that these can be attributed to the cooperation of EGF signalling with the TGF-β signalling pathway and alteration of integrin signalling [39-41].

Despite reports implicating the HER2-HER3 heterodimer as the most potent and mitogenic dimer pair [36-38], in our model, HER3 or HER2/3 overexpression had a less pronounced effect on epithelial architecture, suggesting that the HER2-HER3 heterodimer was not the most potent inducer of aberrant epithelial architecture. Previous reports have demonstrated that the HER2-EGFR heterodimer is also strongly mitogenic and can drive cell migration [42, 43].

The effect of HER overexpression on luminal epithelial architecture has not been studied previously in the presence of both myoepithelial cells and fibroblasts. Here, we illustrated that myoepithelial cells in co-culture with HB2 cells appeared to diminish the disruptive effects of HER overexpression, akin to their recognised tumour suppressive role. On the other hand, co-culture with fibroblasts appeared to promote the opposite effect.

In summary we have successfully produced a robust heterotypic 3D *in vitro* model of normal human breast tissue that incorporated the three major cell types found in the breast in a physiologically relevant matrix of collagen I. We demonstrated individual cell populations were traceable, and formed co-units which shared morphology and phenotype with breast acini of the normal human mammary gland. Phenotypically, HER2 overexpressing tri-cultures had a strikingly similar phenotype to DCIS. Features such as elongated shape, distorted lumens, the presence of discohesive cells and necrosis were all recapitulated upon overexpression of HER2 in the model. The ability to produce structures that reflect early pre-malignant lesions such as DCIS in a physiologically relevant context validated the suitability of our model for cancer initiation studies. The model is robust, amenable to genetic manipulation and can be analysed and quantified by standard laboratory techniques. Our *in vitro* model could be used to dissect the mechanisms behind the early stages of breast cancer initiation.

## MATERIALS AND METHODS

### Tissue

Reduction mammoplasty samples were collected with informed consent via the Leeds Breast Cancer Campaign Tissue Bank following ethical approval (REC: 09/H1306/108, 10/H0308/48).

### Cell culture

The non-tumorigenic breast epithelial cell line, HB2, was used [44]. The myoepithelial cell line Myo1089 was derived from breast reduction mammoplasty tissue and transduced with SV40 large T antigen [2] (gift from Professor Mike O'Hare, Ludwig Institute, London, UK). HB2 cells overexpressing HER2 and HER3 were generated through transfection of pSV2-HER2 [45] or retroviral transduction of pLXSN-ErbB3 [46], followed by single-stain or double-stain fluorescence activated cell sorting (FACS) for HER2 and HER3 or HER2/3 overexpressing cells, respectively to isolate >95% pure cell populations. Fibroblasts were isolated from breast reduction mammoplasty tissue [47] and retrovirally transduced with pBABE-neo-hTERT [48] to increase longevity. Quarterly mycoplasma tests were consistently negative. With the exception of fibroblasts isolated in-house, all cells were Short Tandem Repeat profiled annually by the Leeds Institutes of Molecular Medicine Genomics facility confirming no cross-contamination (last tested April 2014).

### Western blotting

This was performed as previously [47] using NuPAGE® Novex® 4-12% Bis-Tris Gels (Invitrogen, Carlsbad, CA, USA) and proteins transferred to polyvinylidene difluoride membrane (Amersham Biosciences, Buckinghamshire, UK). Membranes were incubated (4°C overnight) with HER2 IgG (Rabbit Polyclonal, Cell Signalling Technology, dilution 1:1000), HER3 IgG (Mouse monoclonal, Clone 2F12, Millipore, dilution 1:1000) or β-actin IgG (Clone AC-15, Sigma, dilution 1:10000) and HRP conjugated secondary antibodies (Santa Cruz Biotechnology, Santa Cruz, USA; dilution 1:10000; 1 hr) before visualisation with SuperSignal West Pico Chemiluminescent Substrate (Thermo Scientific, Loughborough, UK).

### 3D collagen gel culture

Myo1089 cells and fibroblasts were fluorescently labelled with pGIPZ-tGFP and pFURW-dsRed lentiviral vectors respectively and generated using a 2^nd^ generation lentiviral packaging system [49] to enable tracking in culture (Suppl. Figure 1) using immunohistochemistry. Rat Tail Collagen I (BD Biosciences, USA) was diluted with Dulbecco's Phospho Buffered Saline (DPBS, Invitrogen, Carlsbad, CA, USA; 2mg/mL [2] and mixed with eight parts of collagen solution (2mg/mL) to one part 10x Hanks Buffered Salt Solution (HBSS, Sigma, Poole, UK)). The gel was neutralised by drop-wise addition of NaOH until a pink-orange colour was achieved. 3D gel culture medium was prepared (50% complete HAMF12 Nutrient Mixture (Sigma, Poole, UK); 50% complete Dulbecco's modified Eagle's medium (DMEM, Invitrogen, Carlsbad, CA, USA)). Myo1089, HB2 and fibroblast cells were mixed (6:2:1, respectively) in one volume of the above media to achieve a cell density of 4×10^5^ cells /mL gel. This was determined empirically and the ratio which best represented normal breast tissue phenotype (judged by AMH, a specialist breast histopathologist), was selected. 200μl of the gel-cell mixture was added to 12-well plate Transwell® cell culture inserts with PET membranes and 8.0μm pore size (BD Falcon®, USA) and placed within wells of a culture plate (Costar, Corning Inc, Corning, USA). Gels were allowed to solidify (20 mins 37°C in 5% CO_2_) before adding 500μl of media to the top of each gel and 1mL to the bottom of each well in the cell culture plate. Gels were cultured for 21 days with 50% of the media being replaced every three days during this time. Gels were fixed in 10% formalin (Sigma, Poole, UK) for 5 hours, processed (Leica ASP 200 tissue processor), paraffin embedded and 5μm sections taken using a Leica RM2235 microtome (both Leica Microsystems, Milton Keynes, UK) and mounted onto Superfrost Plus slides (BDH, Poole, UK). All slides were H&E stained allowing visualisation.

### Immunohistochemistry

This was performed on 5μm sections from reduction mammoplasty tissue and 3D gels. Primary antibodies included: vimentin (Clone Vim 3B4, 1:200), epithelial membrane antigen (EMA; Clone E29, 1:5000), α-smooth muscle actin (αSMA; Clone 1A4, 1:200), E-cadherin (Clone NCH-38, 1:100), Ki67 (Clone MIB1, 1:1000) [all Dako, Glostrup, Denmark], collagen IV (Clone COL-94, 1:100, Sigma, Poole, UK], M30 (mouse anti-human monoclonal, Clone M30, 1:50, Roche Diagnostics, West Sussex, UK), turboGFP (rabbit polyclonal, 1:500, Pierce, Northumberland, UK) and dsRed (rabbit polyclonal, 1:200, Clontech Laboratories Inc, CA, USA). Primary antibody omission served as negative controls in each run. Antibodies were incubated overnight at 4°C with the exception of turboGFP and dsRed (2 hours, room temperature). Heat-induced antigen retrieval was used with the exception of those incubated with collagen IV which required proteolytic antigen retrieval. Here, sections were incubated at 37°C for 20 mins in 20μg/mL Proteinase K (Fungal, Invitrogen, Carlsbad, CA, USA) in Phosphate Buffered Saline (PBS) prior to blocking. An extra blocking step was used prior to antibody incubation; sections were rinsed twice with 2% Bovine Serum Albumin (BSA, Sigma, Poole, UK) in PBS before adding 100μl 20% goat serum (Dako, Glostrup, Denmark) in PBS (incubation at room temperature for 20 mins).

### Image acquisition

Slides were scanned (Aperio CS and AT scanners; Aperio Technologies Inc, Vista, CA, USA) using x20 and x40 objectives producing images with a final resolution of 0.23 and 0.43μm per pixel respectively. Digital images were acquired using Aperio Imagescope software version 11.

### Quantification of 3D *in vitro* tri-culture model

Annotation tools were used to outline the perimeters of the whole gel section from digital images at 20x magnification, any holes within the gel section and around HB2 units were excluded manually using a computer equipped with a Wacom Cintiq touchscreen monitor. A viable HB2 unit was defined as a cohesive cluster of four or more cells with discernible nuclei. To ensure that units quantified were comprised of HB2 cells, IHC was performed alongside H&E of the gels to confirm that the structures being annotated did not contain turboGFP or dsRed positive cells. HB2 units on the periphery of the gel section or holes were excluded as they did not represent the majority of HB2 structures within the gels (Suppl. Figure 2). Aperio Imagescope software calculated the number of annotations drawn and area of each annotation in μm^2^. Lumen formation was defined as a space within HB2 units that was enclosed by a continuous chain of cells and was counted manually. Any HB2 units that contained luminal spaces with breaches in the surrounding cells were excluded (Suppl. Figure 2). The area of the gel section was calculated by subtracting the area of holes in the gel from the total area of the gel section. Due to the inherent variation between the size of gel sections and therefore the number of units within each gel, the area of HB2 units were normalised by expressing the area of the unit per μm^2^ of gel. This was calculated by taking the area of each HB2 unit and dividing by the area of the gel section. For each experiment, one section from three replicate gels for each variable was quantified. Raw data was inputted into GraphPad Prism 6 software where the mean and SEM was calculated along with statistical analysis. For comparison of the area, number and lumen formation, unpaired t-test analysis was performed where *p* < 0.05 was considered significant.

## SUPPLEMENTARY MATERIAL FIGURES


