# Supplementary material for: Development and characterisation of a 3D multi-cellular in vitro model of normal human breast: a tool for cancer initiation studies
Source: Oncotarget. 2015 Apr 12;6(15):13731–41. doi: 10.18632/oncotarget.3803 (PMC4537045; doi:10.18632/oncotarget.3803)
Supplement: Supplementary file 1 [file oncotarget-06-13731-s001.pdf]

Development and characterisation of a 3D multi-cellular *in vitro* model of normal human breast: a tool for cancer initiation studies

Supplementary Material

Supplementary Figure 1

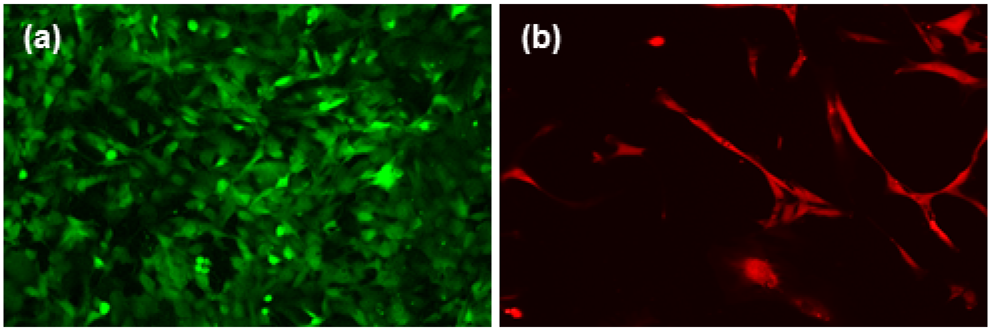

Myo1089 cells (a) and normal human breast fibroblasts (b) were fluorescently labelled with lentivirus. Fluorescence microscopy shows >99% GFP positivity of GFP-labelled Myo1089 cells and >99% dsRed positivity of dsRed-labelled fibroblasts following lentiviral transduction. Original magnification x10.

Supplementary Figure 2

(a)

(b)

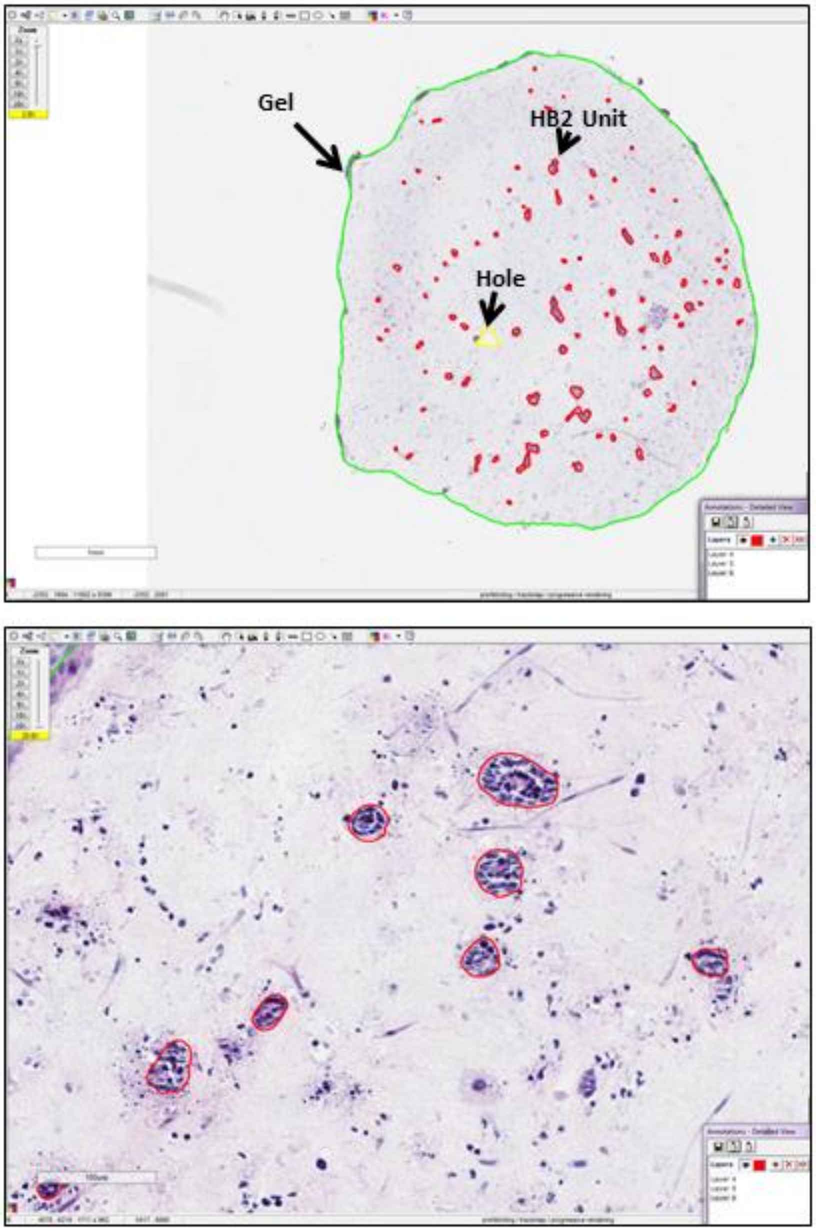

Quantification of HB2 units in 3D *in vitro* culture using Imagescope software

a) Example image showing representative annotations drawn to calculate the total area of each gel section (gel = green), any holes within gel sections (hole = yellow) and of HB2 cell units (HB2 co-unit = Red).

b) Example image at 20x magnification showing representative annotations drawn around HB2 co-units.

Supplementary Figure 3

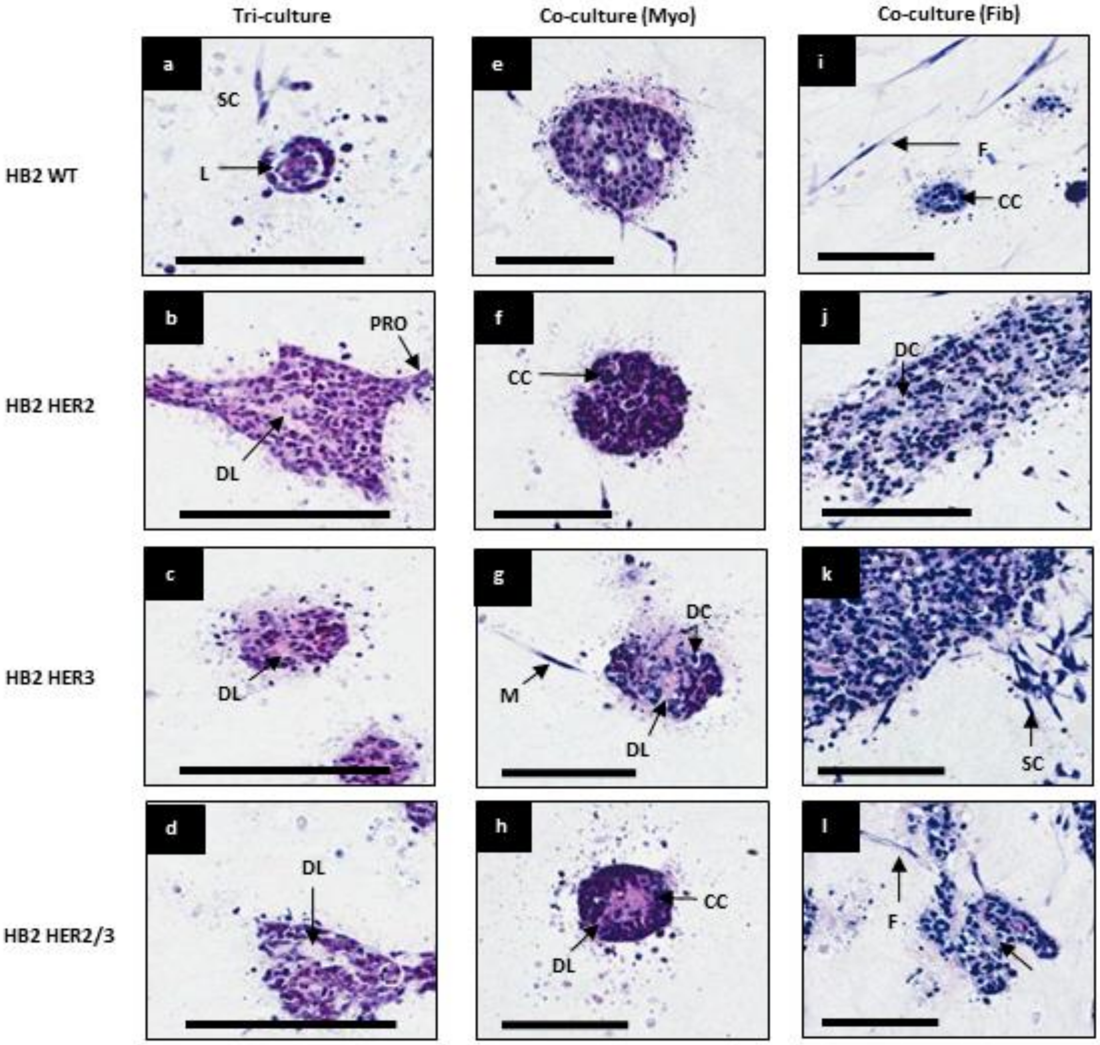

Comparative effects of HER overexpression in different co-culture contexts

Morphologies of HER overexpressing HB2 cells co-cultured with myoepithelial cells (co-culture (Myo)) or fibroblasts (Co-culture (Fibs)) were compared to tri-cultures (a-d). The effects of overexpression of HER proteins had different effects on HB2 unit architecture dependent on the co-culture context. On the whole, myoepithelial cells had an organisational effect on HB2 units inducing formation of rounded and cohesive units regardless of HER protein overexpression (e-h). Co-culture with fibroblasts had opposing effects on HER overexpressing HB2 units. Here, co-units appeared much larger, discohesive and disorganised (i-l). Abbreviations: SC = Spindle cell; L = Lumen; DL = Distorted Lumen; CC = Cohesive Cells; DC = Discohesive cells; M = Myoepithelial cells; F = Fibroblasts. Original magnification = 20x; scale bars = 100µm

Supplementary Figure 4

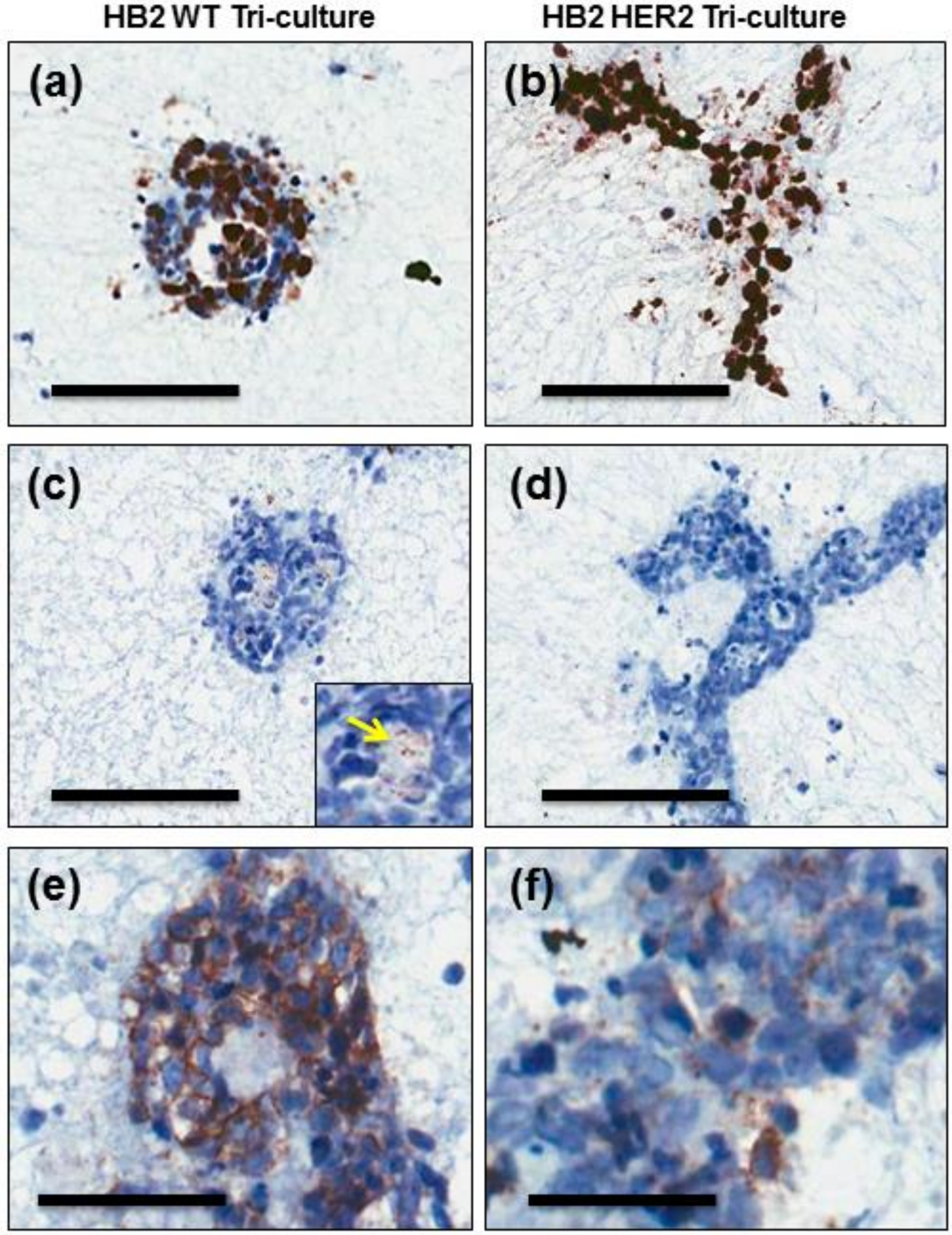

Characterisation of HER2 overexpressing tri-cultures

Proliferation, apoptosis and cell-cell adhesion was assessed by immunohistochemistry in the HB2 HER2 overexpressing DCIS model (b, d, f) and compared to HB2 WT (a, c, e). Both WT and HER2 overexpressing HB2 cells expressed high levels of Ki67 (a, b) while only HB2 WT cells displayed evidence of apoptosis with positive M30 staining within spheroids (arrow, c). Both WT and HER2 overexpressing HB2 cells expressed E-cadherin at cell junctions (e,f) but this was markedly reduced in HER2 overexpressing HB2 cells. Original magnification a-d = 20x; scale bars = 100µm, original magnification e-f = 40x; scale bars = 50µm.
